# Living with diabetes: quality of life and functional impairment among adults with type 2 diabetes in Northern Brazil

**DOI:** 10.1038/s41598-026-37453-7

**Published:** 2026-05-05

**Authors:** Joana Marcela Sales de Lucena, Denise Maria Martins Vancea, Jorge Luiz de Brito-Gomes, Sacha Clael, Alexandre Lima de Araújo Ribeiro, Luiz Guilherme Grossi Porto, Wagner Rodrigues Martins

**Affiliations:** 1 Department of Physical Education, Federal University of Pernambuco – UFPE, Recife Campus, Av. Prof. Moraes Rego, 1235 - Cidade Universitária, Recife, Pernambuco Brazil; 2 Higher School of Physical Education – ESEF, University of Pernambuco – UPE, Santo Amaro Campus, Recife, Pernambuco Brazil; 3 Federal University of the São Francisco Valley – UNIVASF, Petrolina, Pernambuco Brazil; 4 University of Brazilia – UnB, Brasília, Federal District, Brazil; 5 Postgraduate Program in Physical Education – PPGEF, Brasília, Federal District, Brazil

**Keywords:** Type 2 diabetes mellitus, Health-related quality of life, Functional capacity, Glycemic control, Self-care, Rural health, Diseases, Endocrinology, Health care, Medical research

## Abstract

Type 2 diabetes mellitus (T2DM) is a growing public health concern in Brazil, especially in underserved regions where social and geographic barriers may affect disease management and quality of life. Functional capacity and health-related quality of life (HRQoL) are critical dimensions often overlooked in primary care approaches. To assess the functional capacity and HRQoL of individuals with T2DM receiving care in primary health units in Tocantinópolis, Northern Brazil, and to explore associations with sociodemographic and clinical variables. This is a cross-sectional, descriptive, and analytical study involving 99 adults diagnosed with T2DM. Functional capacity was assessed through validated protocols measuring upper and lower limb muscle strength, hamstring flexibility, mobility and cardiorrespiratry fitness. Physical activity was analyzed using the International Physical Activity Questionnaire (IPAQ) and HRQoL was assessed through Diabetes Quality of Life (DQOL). Sociodemographic and clinical data were also collected. Statistical analysis included descriptive statistics and linear regression models. Most participants were women (71%), with a mean age of 58.7 years. The physical and psychological domains of DQOL presented the lowest scores, while the social domain had the highest. Functional capacity was low for most participants. There were significant associations between low physical activity levels and poorer HRQoL scores in the physical and psychological domains. Individuals with T2DM in this sample presented reduced functional capacity and compromised HRQoL, particularly in domains related to physical and psychological well-being. These findings highlight the urgent need for integrated health strategies in primary care that address physical activity and emotional support for people living with diabetes in remote and low-resource settings.

**Supplementary Information:**

The online version contains supplementary material available at 10.1038/s41598-026-37453-7.

## Introduction

Type 2 diabetes mellitus (T2DM) is a chronic disease with a major impact on global health and well-being, ranking among the ten leading causes of death worldwide. In 2024, the International Diabetes Federation (IDF) estimated that over 589 million adults aged 20–79 were living with diabetes—more than 90% of which were type 2 cases. About 60% of diabetes-related deaths occurred among individuals aged 60 years or older, and total health expenditure for this population reached an alarming USD 501.9 billion. In the same year, Brazil ranked sixth globally in diabetes prevalence, with an estimated 16.6 million people aged 20–79 years living with the disease^1^. This is alarming because it highlights the urgent need for preventive strategies and interventions to improve quality of life for people living with T2DM, especially in vulnerable contexts.

T2DM is associated with significant physical, psychological, and social consequences, including microvascular complications (retinopathy, nephropathy, neuropathy) and macrovascular complications (coronary artery disease, cerebrovascular disease, peripheral vascular disease), all of which can severely affect a patient’s daily functioning and well-being^2,3^. Beyond its clinical manifestations, the diagnosis often requires major lifestyle changes—such as dietary adjustments, glucose monitoring, and regular physical activity—which may lead to emotional distress and reduced health-related quality of life (HRQoL).

Quality of life refers to an individual’s perception of well-being across multiple domains, including physical health, social relationships, and emotional state^4^. HRQoL captures how individuals perceive the impact of health condition on their physical, psychological, and social functioning. This perception can influence personal decision-making and guide health professionals in tailoring patient-centered care strategies.

There are few studies assessing the impact of type 2 diabetes mellitus (T2DM) on health-related quality of life (HRQoL) and its relationship with functional capacity^5–7^. Most existing research has examined isolated associations—such as between HRQoL and reduced flexibility, cardiorespiratory fitness, or muscle strength—without analyzing the combined effects of these factors^3,6,8,9^. Considering that HRQoL is multidimensional, assessing the interplay between disease characteristics and physical function may provide a more accurate understanding of how T2DM affects quality of life. In Brazil, as in many low- and middle-income countries, diabetes-related impairments in functional capacity and HRQoL remain under-addressed in primary care, particularly in remote regions with limited access to health services. Therefore, this study aimed to investigate whether functional capacity variables and glycemic control are predictors of HRQoL among individuals with T2DM living in a small town in northern Brazil. Based on previous evidence linking physical performance and metabolic control to perceived well-being we hypothesized that functional capacity variables and glycemic control would be significantly associated with health-related quality of life domains in adults with T2DM in this underserved Brazilian population, contributing novel insights about these relationships in a previously understudied geographic and cultural context.

## Methods

### Type of study

This is a cross-sectional, observational and quantitative predictive study, approved by the Ethics Committee of the Federal University of Tocantins – UFT (CAAE: 59157316.2.0000.5519). All participants were informed about the objectives and procedures of the study and voluntarily agreed to take part. Each participant signed the Informed Consent Form (ICF) prior to data collection; for illiterate participants, the informed consent form was read aloud in full prior to participation, as these individuals had no physical or cognitive impairments and were legally capable, consent was provided directly by the participants, in accordance with the ethical principles established by the Declaration of Helsinki and the Brazilian National Health Council Resolution No. 466/2012.

### Population and sample

The study included adults with type 2 diabetes mellitus (T2DM) registered in primary care units in Tocantins, Brazil. The population of this study included people with type 2 diabetes mellitus treated by the Brazilian Unified Health System (SUS) in Tocantinopolis (TO/Brazil). The resident population is estimated at 23,119 people, 11,200 men of whom 1,107 are aged between 40 and 49 years, and 11,419 women, of whom 1,241 are aged between 40 and 49 years. The city had only 6 basic health units (UBS) and a record of 624 patients, including hypertensive and diabetic patients (with no difference between the two). To calculate the sample size, G-Power^®^ software was used, considering the following parameters: effect size of 0.15; 95% confidence interval; alpha error probability of 0.05; sample power of 80%; partial R^2^ of 0.20, and 10 predictor variables. Sample size was calculated using G-Power^®^ with a minimum of 118 participants. A convenience sampling strategy was adopted considering the small size of the municipality, the limited number of basic health units (six in total), and the relatively low number of individuals diagnosed with type 2 diabetes. To ensure the inclusion of as many eligible participants as possible, researchers accessed the local primary care registry (SUS) and conducted home visits to invite individuals personally. This active recruitment approach allowed the inclusion of participants who might not attend routine appointments at the health units. Inclusion criteria were age over 40 years, a confirmed diagnosis of type 2 diabetes mellitus (T2DM), and registration in the Brazilian Unified Health System (SUS). Exclusion criteria were cognitive impairments or individuals presenting severe physical limitations that could hinder the safe execution of functional tests (e.g., advanced mobility limitations, major limb loss, recent fractures, or severe musculoskeletal or neurological impairments) were excluded from the study.

In total, 100 people with DM2 participated in the collection with the questionnaire, which corresponds to 16% of the total population of the city and 32 (5%) had complete data (questionnaire, blood collection, and functional tests).

### Instruments and variables

Participants were interviewed using a structured questionnaire (25–30 min) covering sociodemographic data, health perception, physical activity (IPAQ, long version), and quality of life using the Diabetes Quality of Life questionnaire (DQOL), which was translated, adapted and validated for the Brazilian population and showing good psychometric qualities^10^ (Cronbach’s α = 0.92). Physical activity was analyzed as a categorical variable using the International Physical Activity Questionnaire (IPAQ). Participants were categorized as inactive (no reported physical activity), minimally active (some activity but not meeting World Health Organization recommendations), or active (≥ 150 min/week of moderate-intensity physical activity). These categories were analyzed in relation to body mass index (BMI) classifications, as shown in Fig. 3. The Diabetes Quality of Life (DQOL) questionnaire includes 46 items across four domains: satisfaction, impact, social/vocational concerns, and diabetes-related concerns. Responses were rated on a 5-point Likert scale, with the satisfaction domain using an intensity scale and the impact and concerns domains using a frequency scale. Overall and domain-specific scores were calculated according to the method proposed by Jacobson et al. (1994)^11^ and transformed to a 0–100 scale, where lower scores indicate poorer HRQoL and higher scores (closer to 100) indicate better perceived quality of life. Additional tests included anthropometry, blood pressure, and HbA1c.

Functional capacity was assessed through validated protocols measuring upper and lower limb muscle strength, hamstring flexibility, mobility (Timed Up and Go test), and cardiorespiratory fitness (6-minute walk). Upper and lower limb strength were measured using the 30-second sit-to-stand test and handgrip test, respectively, according to the ACSM^12^. Cardiorespiratory fitness was assessed using the 6-minute walk test. The distance walked was compared with age- and sex-adjusted normative data and classified as below average, average, or above average^13^. Mobility was evaluated using the Timed Up and Go test, with results categorized as normal mobility (< 10 s), mild mobility impairment (10–20 s), or significant mobility impairment (> 20 s)^14^. Reference values for each test were presented in Table 1, derived from these standardized sources^15^. Glycemic control was determined using fasting glycated hemoglobin (HbA1c) levels and categorical levels were used: good (< 7%), moderate (7–8%), and poor (> 8%) glycemic control, based on ADA criteria.

**Table 1:** Physical characteristics of Brazilians with type 2 diabetes, in 2018.

Variables		Sample				
	Minimum	Mean	Median	Maximum	Standard deviation	Reference values
DM2 diagnosis time (months)	1.00	79.62	72.00	360.00	48.51	-
Systolic pressure (mmHg)	98.60	131.39	129.80	197.60	17.50	120
Diastolic pressure (mmHg)	56.00	78.53	79.45	107.30	9.95	80
Heart rate (bpm)	52.70	77.95	79.00	150.00	12.17	-
Height (m)	1.40	1.58	1.58	1.78	0.09	-
Body mass (kg)	33.90	68.91	66.65	101.90	11.47	-
Abdominal circumference (cm)	66.50	95.25	97.00	119.70	9.65	88 (women) 102 (men)
Fat percentage (%)	16.70	32.06	31.55	52.20	8.81	-
Upper limb strength (Kg)	11.20	21.45	21.20	42.90	6.59	11.6
Lower limb strength (repetitions/30 seconds)	2.00	9.76	10.00	14.70	2.06	12.0
Flexibility (cm)	6.00	23.25	24.00	44.60	10.10	10.0
Mobility (seconds)	14.00	26.21	25.00	45.00	7.50	7.7
Cardiorespiratory fitness (meters)	0.00	433.75	414.50	690.00	106.28	463.6
Capillary blood glucose (mg/dL)	71.00	237.13	230.50	546.00	104.14	-
Glycated hemoglobin (mg/dL)	4.60	8.69	8.20	17.00	2.40	6.5% to 7%

mmHg: millimeters of mercury; bpm: beats per minute (heart rate); m: meter. Kg: kilogram; Cm: centimeter; mg/dL: milligrams per deciliter. *Reference values obtained from previously published studies.*

Data were collected by three trained undergraduate research assistants from the university’s Physical Education Department. All received standardized instruction to ensure uniform interview and testing procedures, including communication, ethics, and measurement protocols. Participants were interviewed individually at home or at the nearest health unit using a structured questionnaire. After the interview, they were invited to attend the university (UFNT) on a scheduled day to perform the functional capacity tests. To minimize observer bias, researchers followed standardized scripts, recorded data immediately after assessments, and were not informed about the study hypothesis. As this was an observational cross-sectional study, blinding of participants was not applicable.

### Statistical analysis

Descriptive statistics (mean, SD, frequencies) and linear regression models were used to evaluate the association between HRQoL domains and independent variables (e.g., HbA1c, functional capacity). Assumptions were tested via Kolmogorov-Smirnov, VIF, Breusch-Pagan, and Durbin-Watson tests. Effect sizes were calculated using Cohen’s f2. Variables with p-values ≤ 0.20 were retained in the final model, as recommended for exploratory regression models^16^, and those with a p-value greater than this were removed by the *Backward* method. Analyses were conducted in R 3.6.2 and Excel. The dataset supporting the findings of this study is publicly available on Zenodo at the following DOI: 10.5281/zenodo.17409351. Detailed procedures for model validation and predictive equations are available in the Supplementary Material.

## Results

### Descriptive results

**Fig. 1:**
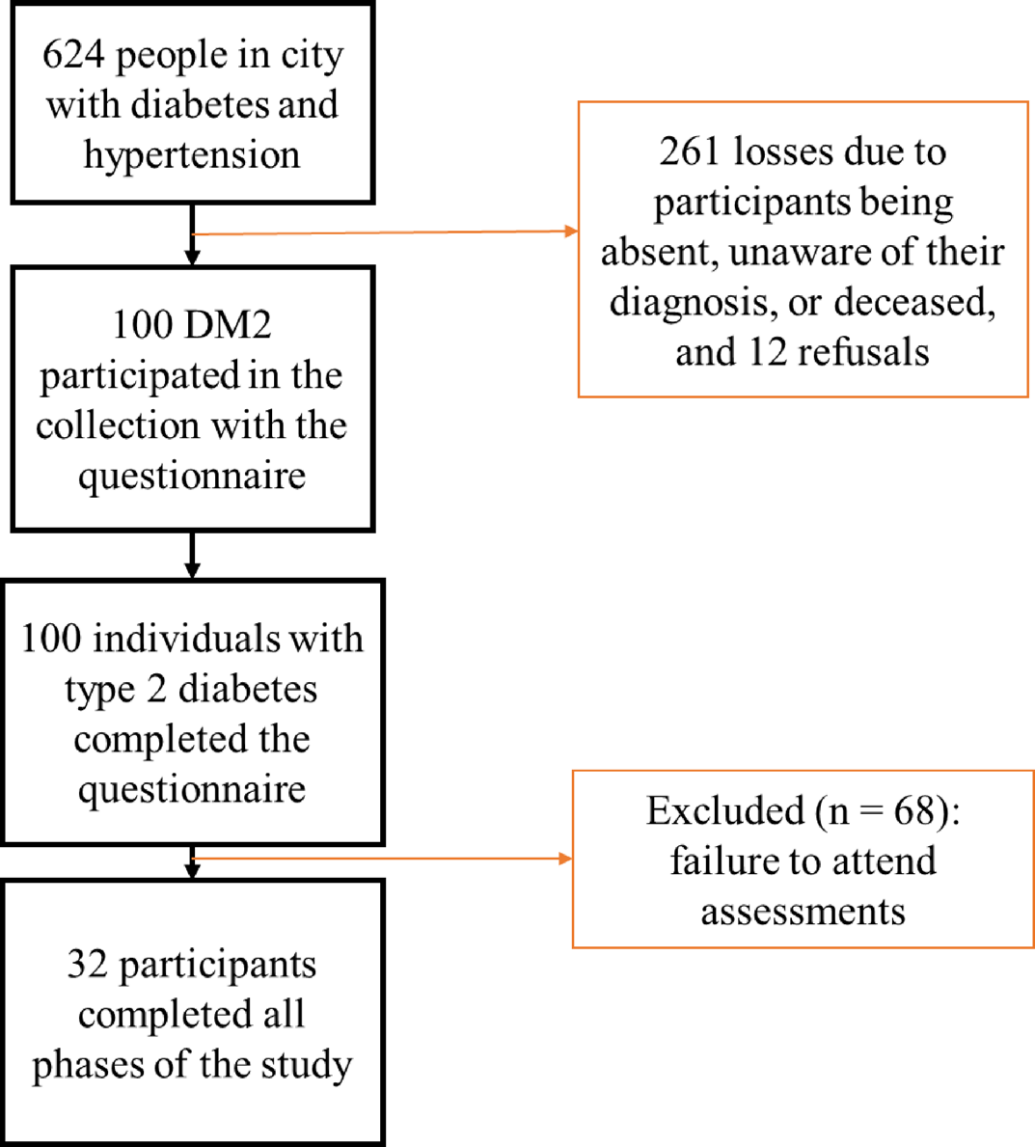
Selection of the sample of Brazilians with type 2 diabetes, in 2018. DM2: type 2 diabetes.

Considering a population of 624 people with DM2 and hypertensive patients treated at Basic Health Units in the city, 373 visits were carried out, of which 261 were considered losses (people who were absent, who ignored the fact of having diabetes, or who had already died at the time of the visit by the data collection team), and 12 refusals. In total, 100 people with DM2 participated in the collection with the questionnaire, which corresponds to 16% of the total population of the city and 32 (5%) had complete data (questionnaire, blood collection, and functional tests) (Fig. 1).

Considering the results presented in Table 2, were female (67.7%), 56% of respondents are married, and the majority have non-white skin color and incomplete high school (88.89% are literate). In addition, the mean age of respondents is approximately 64 years, more than 50% of respondents are lower-middle socioeconomic status (SES), and the mean BMI of respondents is approximately 27 kg/m^2^. Not all had complete data, so the amount of variables changed in each variable.

**Table 2:** Sociodemographic characteristics of Brazilians with DM2, in 2018.

Variables	Sample		
		*n*	%
**Sex**	Female	67	67.68%
	Male	32	32.32%
	Total	99	100
**Skin color**	White	21	22.11%
	Not white	74	77.89%
	Total	95	100
**Schooling**	Illiterate	11	11.22%
	Elementary*	57	58.16%
	High school*	16	16.33%
	Higher*	14	14.29%
	Total	98	100
**Socioeconomic status**	Class A/B	13	14.94%
	Class C	47	54.03%
	Class D/E	27	31.03%
	Total	87	100
		**Mean**	**SD**
**Age (years)**		64.15	11.88
**Income (reais/month)**		1085.58	385.65
**BMI (kg/m** ^**2**^ **)**		27.47	4.33

* complete and incomplete. BMI: body mass index. SD: standard deviation.

Table 1 shows the physical characteristics of the sample. The mean time of diagnosis of diabetes mellitus was approximately 6 years, the mean systolic pressure was approximately 130 mmHg, and the mean diastolic pressure of respondents was 79.5 mmHg. As for the anthropometric characteristics, the mean abdominal circumference was approximately 95 centimeters, indicating a high value and a greater risk of cardiovascular events^17^; the mean body mass of the interviewees was approximately 68 kg and the average fat percentage of the individuals was 32.06%. Regarding the variables related to functional capacity, the average strength of the upper limbs was 21.45 kgf, considered a high value for the mean age^15^; on the other hand, lower limb strength had a mean of nine repetitions per 30 s, which can be considered a lower value than expected^15^. The mean flexibility was 23.25 cm, considered a very good mean flexibility for the age group of this sample^12^. The mobility of the group, measured by the TUG, presented a mean time of 26 s, a value classified as a moderate risk of falls^13^, while cardiorespiratory fitness had an average of approximately 400 m in 6 min, which can be considered a value below the expected for the mean age^15^. Regarding glycemic control, the mean capillary blood glucose was 237 mg/dL and the mean glycated hemoglobin was 8.84 mg/dL (Table 1), these values are above those recommended for people with DM2, which are 115 mg/dL and 7% for fasting glucose and glycated hemoglobin, respectively^1^.

**Fig. 2:**
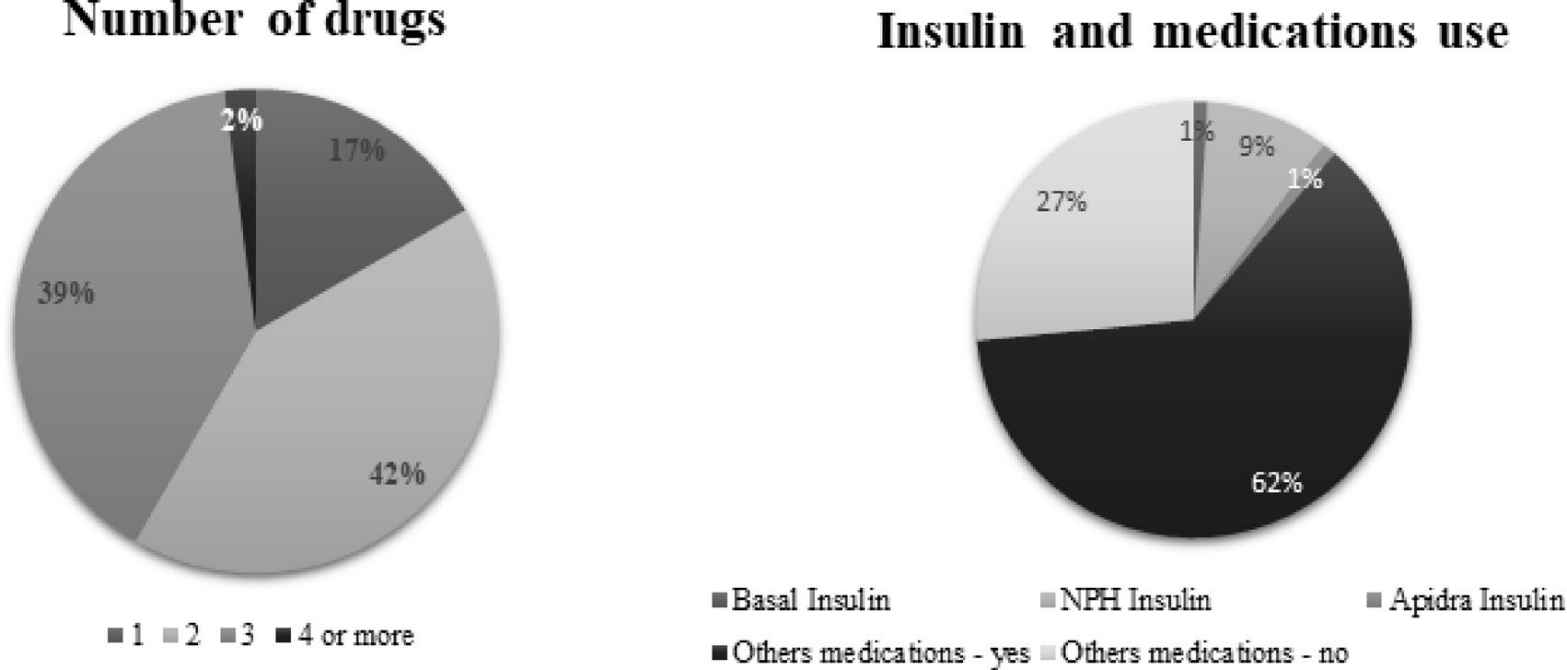
Medications and insuline use in Brazilians with type 2 diabetes, in 2018.

As shown in Fig. 2, approximately 40% used at least 2 medications to control diabetes, only nine participants used insulin, 70% reported using other medications that were not for DM2, and almost 80% of the sample reported having some type of DM2 complication. However, when asked specifically about which complication (eye, kidney, heart, or foot problems) the majority reported not having the complication or any other health problem, with the exception of hypertension, which was the most commonly reported complication among the participants (63.54%) (Table 3). These data are important because they emphasize the importance of information for the patient during the consultations, as considering that the DM2 diagnosis time had an average of six years, it is possible that the patients already have some complications, but have no knowledge about them or have not been informed about them by the doctor.

**Fig. 3:**
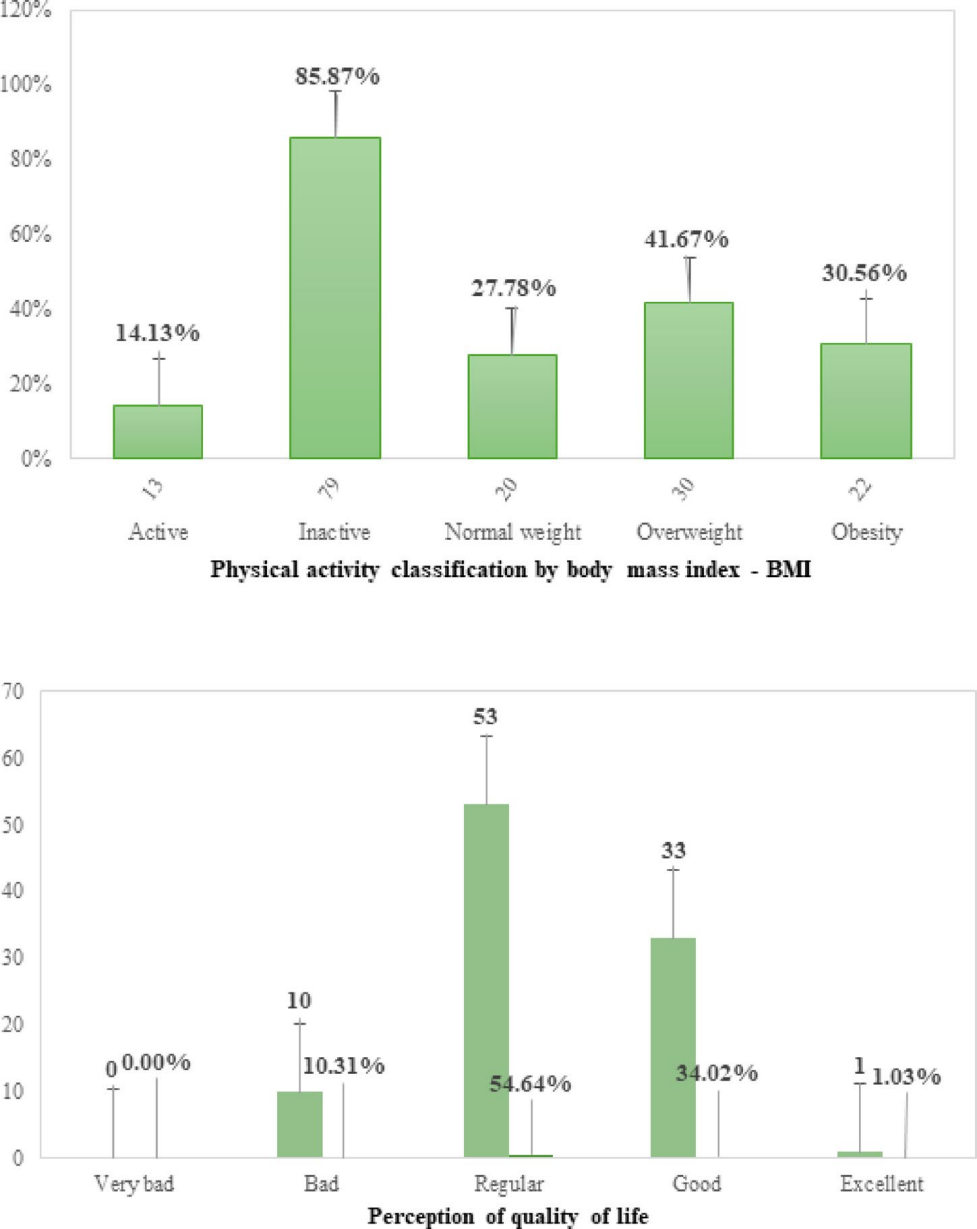
Classification of physical activity, body mass index and quality of life of Brazilians with type 2 diabetes, in 2018.

**Table 3:** Characteristics of chronic complications of Brazilians with type 2 diabetes, in 2018.

Variables		Sample	
	Items	*n*	%
**Self-reported complications**	Yes	76	79.17%
	No	20	20.83%
Retinopathy	Yes	40	41.67%
	No	56	58.33%
Diabetic foot	Yes	5	5.21%
	No	91	94.79%
Nephropathy	Yes	17	17.71%
	No	79	82.29%
Hypertension	Yes	61	63.54%
	No	35	36.46%
Heart disease	Yes	12	12.50%
	No	84	87.50%
Neuropathy	Yes	11	11.46%
	No	85	88.54%
Other health problems	Yes	4	4.17%
	No	92	95.83%

In Fig. 3, approximately 41% of the participants are overweight and most of the participants were classified as physically inactive. Regarding the assessment of quality of life, and perception of their sleep health status, almost all participants perceived all these aspects as “regular”.

**Fig. 4:**
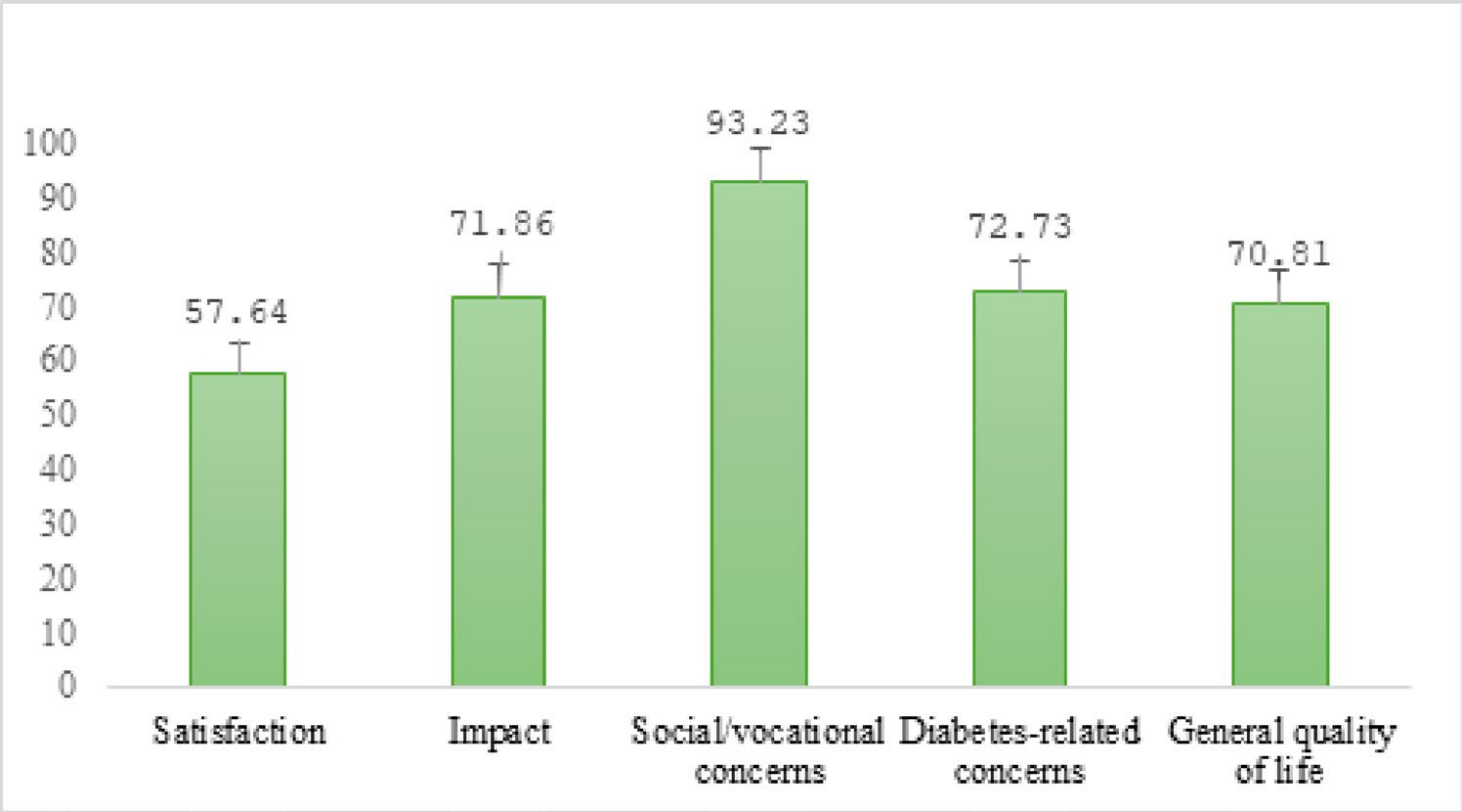
Score of the domains of health-related quality of life and their effects in the sample of Brazilians with type 2 diabetes, in 2018.

The HRQoL of the sample is presented in Fig. 4. The data show that the mean score of the general quality of life was 70.8 points, which indicates a high HRQoL, from a score of 0 to 100 points. Regarding domains, the social/vocational concerns domain received the highest score, followed by diabetes-related concerns and diabetes impact. The domain with the lowest score, indicating the lowest HRQoL, was the Satisfaction with treatment domain.

With respect to the association between HRQoL, DM2 characteristics, variables related to functional capacity, and glycemic control, Table 4 presents the linear regression models for each domain and the overall HRQoL score. Thirty-two observations that had complete data to compose the multiple linear regression model were considered for analysis. Considering the final sample size, the Effect Size was calculated using the Cohen test, and the result indicated an effect size of 1.09, considered high for the sample^18^.

**Table 4:** Linear regression models (baseline and adjusted) between Health-Related quality of life (HRQoL) and independent variables of Brazilians with type 2 diabetes, in 2018.

Dependent variables	Independent variables	β	Standard error	t-value	*p*
**1. Satisfaction with treatment**					
**Base model (R**^**2**^ **= 11.8%)**	Intercept	−109.783	64.379	−1.705	0.102 *
	DTDM2 (years)	−0.907	1.118	−0.811	0.426
	No complications from DM2	3.785	7.241	0.523	0.606
	Strength of upper limbs (Kg)	0.273	0.690	0.397	0.695
	Strength of lower limbs (rep.)	3.122	1.499	2.082	0.049 *
	Flexibility (cm)	0.271	0.287	0.945	0.355
	Mobility (seconds)	2.200	1.070	2.056	0.051 *
	Cardiorespiratory fitness (m)	0.161	0.084	1.914	0.068 *
	HbA1c (%)	0.195	1.383	0.141	0.889
**Final model** **(R**^**2**^ **= 12.5%)** **(ES = 1.09)**	Intercept	21.596	14.551	1.484	0.149 *
	No complications from DM2	7.878	5.955	1.323	0.197 *
	Strength of upper limbs (Kg)	2.489	1.342	1.855	0.074 *
	Strength of lower limbs (rep.)	0.360	0.276	1.303	0.200 *
**2. Impact of Diabetes**					
**Base model** **(R**^**2**^ **= 0.8%)**	Intercept	34.366	62.434	0.550	0.587
	DTDM2 (years)	0.511	1.084	0.471	0.642
	No complications from DM2	0.186	7.023	0.027	0.979
	Strength of upper limbs (Kg)	−0.422	0.669	−0.632	0.534
	Strength of lower limbs (rep.)	2.476	1.454	1.703	0.102 *
	Flexibility (cm)	0.233	0.278	0.839	0.410
	Mobility (seconds)	0.550	1.038	0.531	0.601
	Cardiorespiratory fitness (m)	0.052	0.082	0.638	0.530
	HbA1c (%)	−2.332	1.341	−1.739	0.095 *
**Final model**	Intercept	72.475	16.245	4.461	< 0.001 *
**(R**^**2**^ **= 14.4%)**	Strength of lower limbs (rep.)	2.104	1.216	1.731	0.094 *
**(ES = 1.07)**	HbA1c (%)	−2.109	1.122	−1.880	0.070 *
**3. Social/vocational concerns**	Intercept				
**(R**^**2**^ **= 14.3%)**	DTDM2 (years)	0.829	0.557	1.489	0.150 *
	No complications from DM2	7.066	3.604	1.960	0.062 *
	Strength of upper limbs (Kg)	0.183	0.343	0.533	0.599
	Strength of lower limbs (rep.)	0.652	0.746	0.874	0.391
	Flexibility (cm)	0.296	0.143	2.075	0.049 *
	Mobility (seconds)	−0.605	0.533	−1.135	0.268
	Cardiorespiratory fitness (m)	−0.077	0.042	−1.828	0.080 *
	HbA1c (%)	−1.435	0.688	−2.086	0.048 *
**Final model** **(R**^**2**^ **= 0.125%)** **(ES = 0.14)**	Intercept	96.261	6.893	13.966	< 0.001 *
	Flexibility (cm)	0.260	0.139	1.872	0.071 *
	HbA1c (%)	−0.955	0.626	−1.525	0.138 *
**4. Diabetes-related concerns** **(R**^**2**^ **= − 0.044%)**	Intercept	−18.018	98.268	−0.183	0.856
	DTDM2 (years)	2.106	1.707	1.234	0.230
	No complications from DM2	14.331	11.053	1.297	0.208
	Strength of upper limbs (Kg)	0.550	1.053	0.522	0.607
	Strength of lower limbs (rep.)	2.736	2.289	1.196	0.244
	Flexibility (cm)	0.219	0.438	0.501	0.621
	Mobility (seconds)	1.521	1.633	0.931	0.361
	Cardiorespiratory fitness (m)	0.041	0.128	0.317	0.754
	HbA1c (%)	−2.950	2.110	−1.398	0.176 *
Final model*	-	-	-	-	-
**5. Overall HRQoL score**					
**Base model** **(R**^**2**^ **= 11.8%)**	Intercept	−2.448	49.861	−0.049	0.961
	DTDM2 (years)	0.236	0.866	0.272	0.788
	No complications from DM2	3.637	5.608	0.648	0.523
	Strength of upper limbs (Kg)	−0.019	0.534	−0.035	0.972
	Strength of lower limbs (rep.)	2.432	1.161	2.094	0.048 *
	Flexibility (cm)	0.254	0.222	1.143	0.265
	Mobility (seconds)	0.997	0.829	1.203	0.241
	Cardiorespiratory fitness (m)	0.067	0.065	1.029	0.314
	HbA1c (%)	−1.425	1.071	−1.331	0.196 *
**Final model** **(R**^**2**^ **= 12.5%)**	Intercept	53.617	14.777	3.628	0.001 *
**(ES = 1.09)**	DTDM2 (years)	0.524	0.724	0.723	0.476
	Strength of lower limbs (rep.)	1.932	1.013	1.906	0.067 *
	Flexibility (cm)	0.318	0.210	1.513	0.142 *
	HbA1c (%)	−1.307	0.990	−1.321	0.198 *

* Baseline models included diabetes diagnosis time (DTDM2), presence of diabetes-related complications, upper and lower limb muscle strength, flexibility, mobility (Timed Up and Go), cardiorespiratory fitness (6-minute walk test), and glycated hemoglobin (HbA1c). Final adjusted models retained covariates meeting predefined selection criteria (*p* ≤ 0.20) and satisfying linear regression assumptions. Model-specific covariate composition varied according to outcome domain. Additional details on model building procedures and diagnostic analyses are provided in the Supplementary Material. N: Newtons; DTDM2: DM2 diagnosis time; rep: repetitions for 30 s; m: meters; kg: kilogram; cm: centimeter; m: meter; HbA1c (%) – glycated hemoglobin percentage. ES: effect size according to the calculation of Cohen’s f^2^ value. Total observations in each model (n): 32 participants.

Observing the results of Table 4, several variables placed in the model were significant, considering a 20% level of significance. Below, five multiple linear regression models will be presented, covering the associations between HRQoL and its respective domains, the characteristics of DM2, the variables related to functional capacity, and glycemic control.

- *Satisfaction with treatment domain*

Model 1 analyzed the associations between independent variables and satisfaction with treatment domain. Not having DM2 complications (β = 7.9; *p* = 0.197), upper limb strength (β = 2.5; *p* = 0.074), and lower limb strength (β = 0.360; *p* = 0.2) were associated with this domain. The adjusted model showed that these variables predict 12.5% of the HRQoL satisfaction with treatment domain. Participants who reported no complications had seven points more on the HRQoL than those who had some type of complication resulting from DM2. In addition, those with more strength in the upper and lower limbs also had a better perception of satisfaction with the treatment (Table 4).

- *Impact of diabetes mellitus* *domain*

Considering the impact of DM2, the results shown in model 2 indicated a positive association with lower limb strength (β = 2.104; *p* = 0.094) and an inverse association with glycated hemoglobin (β = −2.109; *p* = 0.070). The final model explained 14.4% (R^2^) of the variation in the dependent variable. With each addition of one unit in the sit to stand test, the HRQoL score increased by 2.104 points; whereas with each addition of one unit in glycated hemoglobin, there was a decrease of 2 points in terms of the impact of the disease on the HRQoL.

- *Social/vocational concerns domain*

Model 3 demonstrates the associations of independent variables with the social/vocational concerns domain. Only flexibility had a positive association with this HRQoL domain (β = 0.260; *p* = 0.071), while the association with glycated hemoglobin was inverse (β = −0.955; *p* = 0.138), and R^2^ was 0.125%.

- *Diabetes-related concerns domain*

Model 4 refers to the association between the independent variables and the diabetes-related concerns domain. Initially, the base model was analyzed, but a model was not found in which the independent variables had an influence on the dependent variable and the assumptions of linear regression were met, even though all possible combinations between the variables were tested for the realization of the model, however none of the combinations were significant. Thus, the normal linear model is not suitable for thwaw data.

- *Overall HRQoL score*

Lower limb strength (β = 1.932; *p* = 0.067), flexibility (β = 0.318; *p* = 0.142), and glycated hemoglobin (β = −1.307; *p* = 0.198) were associated with the overall HRQoL score in model 5; this final model predicts 12.5% of the variation in HRQoL. With each increase of one unit in the sit to stand test, the overall quality of life increased by almost two points, and with each increase of one unit in glycated hemoglobin, the general quality of life decreased by 1.307 points.

## Discussion

This study aimed to examine whether characteristics of type 2 diabetes mellitus (T2DM), functional capacity, and glycemic control are predictors of health-related quality of life (HRQoL) among Brazilian adults living with T2DM. Our findings indicate that a shorter duration of diagnosis (less than ten years), absence of diabetes-related complications, greater upper and lower limb strength, and higher levels of flexibility were positively associated with better HRQoL and its domains, including satisfaction with treatment (R2 = 12.5%), impact of diabetes (R2 = 14.4%), social/vocational concerns (R2 = 0.125%), and diabetes-related concerns. Conversely, higher levels of glycated hemoglobin were inversely associated with HRQoL (β = −1.307; *p* = 0.198), suggesting that poor glycemic control may negatively influence individuals’ perception of the disease and their social well-being.

Previous studies have consistently identified several predictors of HRQoL in patients with T2DM, including sex, advanced age, and lower socioeconomic status^7^. Additional negative predictors include sex, insulin use, complex pharmacological treatment^7^, longer disease duration and presence of comorbidities or complications^5,7^. Psychosocial stressors such as limited social support, negative coping mechanisms, and low cognitive appraisal also appear to be associated with diminished HRQoL^19^.

Physical health factors—frequently exacerbated by T2DM—such as elevated body mass index, reduced flexibility^5^, poor cardiorespiratory fitness^20^, diminished muscle strength^2,21^, metabolic syndrome^3^ and fall risk^22^, have also demonstrated negative associations with HRQoL. These findings have been replicated across various regions, including Europe, Asia, and Latin America.

It is important to interpret these findings considering the specific sociodemographic and lifestyle context of the present sample. Participants were predominantly from a lower-middle socioeconomic background, a profile commonly observed among individuals with chronic diseases in public health services in Northeastern Brazil. This context is frequently characterized by limited access to structured physical activity programs, greater occupational and financial instability, and increased psychosocial burden, which may intensify the impact of functional and metabolic impairments on perceived quality of life.

The heterogeneity of instruments used in HRQoL assessment complicates cross-study comparisons. Each tool is grounded in different theoretical frameworks and captures distinct domains, limiting generalizability. The present study addressed this limitation by employing the DQOL, a validated instrument specifically designed for individuals with diabetes and culturally adapted for the Brazilian population^10,11^.

Our data indicates that functional capacity variables exert considerable influence on HRQoL in individuals with T2DM. These relationships are further shaped by sociodemographic and cultural contexts, especially in a country as diverse as Brazil.

### HRQoL and diabetes complications

Absence of chronic complications was associated with an increase of up to seven points in the satisfaction with treatment domain (R2 = 12.5%). Previous research has shown that complications such as retinopathy, nephropathy, and neuropathy triple the likelihood of poor HRQoL^3,5^. This finding supports the notion that awareness of one’s complication status may enhance engagement in preventive behaviors and disease management, generating a positive feedback loop.

Drawing on Orem’s Self-Care Theory^23^, self-care comprises basic capabilities, health knowledge, and action. In T2DM management, self-care is essential for preventing complications and improving HRQoL. In this sample, self-care practices such as medication adherence, dietary regulation, and foot self-examination were relatively common^24,25^. However, practices like blood glucose monitoring and physical activity remain insufficient, indicating gaps in comprehensive self-management.

### HRQoL and muscle strength

Greater upper limb strength was associated with better satisfaction with treatment (R2 = 12.5%), although mean grip strength in the sample was below normative values for the age group^15^. T2DM is known to impair protein metabolism, accelerating muscle loss. This condition, combined with disease duration and age, may lead to sarcopenia, reduced functionality, and lower HRQoL^2,21,26^.

While some studies suggest that improved muscle strength reduces insulin resistance^26^, other studies reinforce the association between worse glycemic and lipid control and low muscle strength^27,28^. In this study, despite reduced strength, participants reported satisfaction with care, possibly reflecting the perceived adequacy of healthcare provided by the Brazilian Unified Health System (SUS).

Lower limb strength also emerged as a key factor influencing the impact of diabetes on HRQoL (R2 = 14.4%) and the overall HRQoL score (R2 = 12.5%). Given the relationship between muscle weakness, decreased mobility, and fall risk^29,30^, promoting muscle strength may mitigate negative health outcomes and enhance well-being.

### HRQoL and flexibility

Flexibility was positively associated with better HRQoL, especially in the social/vocational domain (β = 0.260; *p* = 0.071). Hyperglycemia-induced collagen glycation can increase joint stiffness and contribute to musculoskeletal complications such as tendinopathies and capsulitis^31^. These conditions limit daily function and can affect mental health^22,32^. Despite this, participants in our study demonstrated good flexibility levels, which may help explain their relatively positive HRQoL assessments.

### HRQoL and glycemic control

Elevated HbA1c levels were inversely related to both overall HRQoL and the social/vocational concerns domain (β = −1.307 and − 0.955, respectively). These findings are aligned with prior literature indicating associations between poor glycemic control and cardiovascular risk, increased BMI, insulin resistance, and reduced treatment responsiveness^24,25,30^.

A lifestyle intervention conducted in Houston, USA, led to significant improvements in quality of life and glycemic control. In parallel, a remarkable increase was observed in functional capacity, strength, and cardiorespiratory performance (VO₂peak), along with higher SF-36 physical scores, reflecting a positive impact on perceived health and overall well-being^2^. These results underscore the importance of patient understanding in fostering effective self-care and emotional well-being. The ADA (2000) also identifies the burdens of self-management, symptom vigilance, and fear of complications as key determinants of HRQoL. A bidirectional relationship appears to exist as glycemic control deteriorates, HRQoL worsens; conversely, enhanced well-being may promote adherence and metabolic stability.

### Limitations and strengths

This study presents some limitations that should be considered when interpreting the findings. The cross-sectional design precludes establishing causal relationships between functional capacity, glycemic control, and quality of life. Although data collection procedures were standardized, the use of a convenience sample may introduce selection bias and limit the generalizability of the results to other populations. The sample consisted of adults from a small town in northern Brazil, which may influence external validity due to regional and socioeconomic characteristics. Additionally, self-reported information — particularly regarding clinical history and lifestyle — may be subject to recall or social desirability bias. Nonetheless, the internal validity of the study was strengthened using validated instruments, detailed characterization of a remote Brazilian population, trained data collectors, standardized measurement protocols and the integration of multiple physical and clinical variables. As the first Brazilian investigation to examine the relationship between functional capacity and HRQoL among individuals with T2DM, this study provides foundational insights to inform future research and public health strategies.

## Conclusion

Our hypothesis was partially supported. Disease-related characteristics, including a shorter duration of diagnosis (less than ten years) and the absence of diabetes-related complications, as well as functional capacity variables—such as upper and lower limb strength and flexibility—were positively associated with better health-related quality of life (HRQoL) and its domains, including treatment satisfaction, disease impact, social/vocational concerns, and diabetes-related worries. Conversely, higher glycated hemoglobin levels were inversely associated with HRQoL, suggesting that poor glycemic control may intensify the perceived burden of the disease and related social concerns among individuals with type 2 diabetes. However, due to the cross-sectional design, these findings reflect associations rather than predictive relationships. Future longitudinal studies are warranted to clarify temporal pathways and establish causal and predictive links between functional capacity, glycemic control, and HRQoL.

## Supplementary Information

Below is the link to the electronic supplementary material.

Supplementary Material 1

Supplementary Material 2

## Data Availability

The datasets generated and/or analyzed during the current study are publicly available in the Zenodo repository at: [https://doi.org/10.5281/zenodo.15657420].
