# Supplementary material for: Living with diabetes: quality of life and functional impairment among adults with type 2 diabetes in Northern Brazil
Source: Sci Rep. 2026 May 5;16:20682. doi: 10.1038/s41598-026-37453-7 (PMC13334035; doi:10.1038/s41598-026-37453-7)
Supplement: Supplementary file 1 — Supplementary Material 1 [file 41598_2026_37453_MOESM1_ESM.docx]

**Living with Diabetes: Quality of Life and Functional Impairment among Adults with Type 2 Diabetes in Northern Brazil**

Profª. Drª Joana Marcela Sales de Lucena^1,4*^

Profª. Drª Denise Maria Martins Vancea^2^

Prof. Dr. Jorge Luiz de Brito Gomes^3^

Prof. Dr. Sacha Clael^4,5^

Prof. Me. Alexandre Lima de Araújo Ribeiro ^4,5^

Prof. Dr. Luiz Guilherme Grossi Porto ^4,5^

Prof. Dr. Wagner Rodrigues Martins ^4,5^

**Filiation**

1. Federal University of Pernambuco – UFPE, Recife Campus, Department of Physical Education, **Recife**, Pernambuco, **Brazil**. 2. University of Pernambuco – UPE, Higher School of Physical Education – ESEF, Santo Amaro Campus, **Recife**, Pernambuco, **Brazil**. 3. Federal University of the São Francisco Valley – UNIVASF, **Petrolina**, Pernambuco, **Brazil**. 4. University of Brazilia – UnB, **Brasília**, Federal District, **Brazil.** 5. Postgraduate Program in Physical Education – PPGEF, **Brasília**, Federal District, **Brazil.**

***** **Correspondence author**

Profª. Drª Joana Marcela Sales de Lucena

Av. Prof. Moraes Rego, 1235 - Cidade Universitária, Recife - PE, 50670-901

E-mail: [joana.marcela@ufpe.br](mailto:joana.marcela@ufpe.br)

**Supplementary material**

This file provides additional details regarding the statistical analyses and predictive modeling procedures performed in the study

- **Quality assessment of linear regression models**

To verify the quality of fit of the models, the residuals of each model were generated and the distribution of these residuals in the Envelope Diagram were analyzed. Only two models showed residuals outside the envelope's confidence bands for a tolerance level of 5%. The adjustment was also verified by the Kolmogorov-Smirnov test and all residuals are normal since they presented a p-value greater than 0.05. To assess whether there was a multicollinearity problem in the adjusted model, the values ​​of the VIF statistic (Variance Inflation Factor) were obtained for each independent variable and none presented a value greater than 1.5. Since the value of the VIF statistic must have a value lower than 4, it is concluded that none of the variables present multicollinearity. To verify if the error variance was constant, the Breusch-Pagan test was used, for which the null hypothesis of the test indicates that the error variance is constant. All models presented a p-value greater than 0.05, not rejecting the null hypothesis. To verify if the errors are independent, the Durbin-Watson test was used, in which the null hypothesis of the test is that the correlation between the errors is equal to zero, that is, that the errors are independent, the p-value for this test was greater than 0.50, so the null hypothesis that the errors are independent is not rejected. Thus, it was possible to conclude that the models can be validated.

## Use of predictive model of health-related quality of life

With respect to the association between HRQoL, DM2 characteristics, variables related to functional capacity, and glycemic control, Table 6 presents the linear regression models for each domain and the overall HRQoL score. Thirty-two observations that had complete data to compose the multiple linear regression model were considered for analysis. Considering the final sample size, the Effect Size was calculated using the Cohen test, and the result indicated an effect size of 1.09, considered high for the sample(23).

Observing the results of table 6, several variables placed in the model were significant, considering a 20% level of significance. Below, five multiple linear regression models will be presented, covering the associations between the HRQoL and its respective domains, the characteristics of DM2, the variables related to functional capacity, and glycemic control.

- Satisfaction with treatment domain

Model 1 analyzed the associations between the independent variables and the satisfaction with treatment domain. Not having DM2 complications (β = 7.9; p = 0.197), upper limb strength (β = 2.5; p = 0.074), and lower limb strength (β = 0.360; p = 0.2) were associated with this domain. The adjusted model showed that these variables predict 12.5% of the HRQoL satisfaction with treatment domain. The following predictive model equation was established:

*Y = 21.596 + 7.878 Complication due to diabetes (No) + 2.489** *Handgrip Test + 0.360** *Stand up and sit down test*

Participants who reported no complications had seven points more on the HRQoL than those who had some type of complication resulting from DM2. In addition, those with more strength in the upper and lower limbs also had a better perception of satisfaction with the treatment (Table 6).

- Impact of diabetes mellitus domain

Considering the impact of DM2, the results shown in model 2 indicated a positive association with lower limb strength (β = 2.104; p = 0.094) and an inverse association with glycated hemoglobin (β = -2.109; p = 0.070). The final model explained 14.4% (R^2^) of the variation in the dependent variable. With each addition of one unit in the sit to stand test, the HRQoL score increased by 2.104 points; whereas with each addition of one unit in glycated hemoglobin, there was a decrease of 2 points in terms of the impact of the disease on the HRQoL. For this model, the following predictive equation was designed:

*Y = 72.475 + 2.104* Sit to stand test – 2.109** *Glycated hemoglobin*

- Social/vocational concerns domain

Model 3 demonstrates the associations of independent variables with the social/vocational concerns domain. Only flexibility had a positive association with this HRQoL domain (β = 0.260; p = 0.071), while the association with glycated hemoglobin was inverse (β = -0.955; p = 0.138), and R^2^ was 0.125%. The predictive model was determined by the following equation:

*Y = 96.261 + 0.260*Wells test – 0.955* Glycated hemoglobin*

- Diabetes-related concerns domain

Model 4 refers to the association between the independent variables and the diabetes-related concerns domain. Initially, the base model was analyzed, but a model was not found in which the independent variables had an influence on the dependent variable and the assumptions of linear regression were met, even though all possible combinations between the variables were tested for the realization of the model, however none of the combinations were significant. Thus, the normal linear model is not suitable for thwaw data.

- Overall HRQoL score

Lower limb strength (β = 1.932; p = 0.067), flexibility (β = 0.318; p = 0.142), and glycated hemoglobin (β = -1.307; p = 0.198) were associated with the overall HRQoL score in model 5; this final model predicts 12.5% of the variation in HRQoL. With each increase of one unit in the sit to stand test, the overall quality of life increased by almost two points, and with each increase of one unit in glycated hemoglobin, the general quality of life decreased by 1.307 points. For the general HRQoL predictive model, the following equation was designed:

Y = *53.617* + 0.*524** *How long have you had diabetes (years) + 1.932* Sit to stand test* + 0.*318*Wells test– 1.307* Glycated hemoglobin*

- Use of the equation to predict HRQoL

The HRQoL measurement can be an important tool for assessing the health of patients with DM2 and an indication of their perception of the management of the disease. This evaluation should help guide the treatment, which can focus on physical, emotional, or social aspects, allowing a focus on more complete health. The DQOL enables an overall assessment of HRQoL, using all items. To measure a patient's HRQoL, it is necessary to establish the following characteristics: diagnosis time, sit to stand test result, flexibility, and HbA1c. For example, a patient: with 2 years with DM2, 6 repetitions in the sit to stand test; 20cm in the flexibility test; and 7% glycated hemoglobin -

*Y = 53.617 + 0.524* (2) + 1.932*(6) + 0.318*(20) – 1.307*(0.07)*

*Y = 53.617 + 1.048 + 11.592 + 6.36 – 0.092*

*Y = 72.525*

The result indicates that the HRQoL of this individual has a good score, around 72 points.

Still considering the same patient, it is possible to calculate the impact using only the value of the sit to stand test and HbA1c:

*Y = 72.475 + 2.104* (6) – 2.109 * (0.07)*

*Y = 72.475 +12.624 – 0.14763*

*Y = 72.475 +12.47637*

*Y = 84.95137*

The result indicates that the patient has a high score in the impact domain and, therefore, may need an intervention that considers aspects related to this domain, such as pain, physical limitations, and acceptance of the health condition.
