# Supplementary material for: Living with diabetes: quality of life and functional impairment among adults with type 2 diabetes in Northern Brazil
Source: Sci Rep. 2026 May 5;16:20682. doi: 10.1038/s41598-026-37453-7 (PMC13334035; doi:10.1038/s41598-026-37453-7)
Supplement: Supplementary file 2 — Supplementary Material 2 [file 41598_2026_37453_MOESM2_ESM.pdf]

PARECER CONSUBSTANCIADO DO CEP

DADOS DO PROJETO DE PESQUISA

Título da Pesquisa: Prática de Exercício Físico e Diabetes Mellitus
Pesquisador: Joana Marcela Sales de Lucena
Área Temática:
Versão: 2
CAAE: 59157316.2.0000.5519
Instituição Proponente: Fundação Universidade Federal do Tocantins
Patrocinador Principal: Financiamento Próprio

DADOS DO PARECER

Número do Parecer: 1.857.080

Apresentação do Projeto:
O objetivo deste estudo será analisar o uso de diferentes formas de exercício físico no tratamento do diabetes mellitus de diabéticos tipo 2. Trata-se de um estudo experimental. Para compor a amostra deste estudo serão convidados indivíduos com diabetes tipo 2, ambos os sexos, atendidos pelas Unidades Básicas de Saúde da Cidade de Tocantinópolis. Os participantes preencherão um Protocolo de Inscrição contendo informações sociodemográficas e informações sobre qualidade do sono, percepção de saúde, qualidade de vida relacionada à saúde, informações sobre o diabetes e prática anterior de exercícios físicos antes e depois do Programa de Exercício Físico para Diabéticos. Os critérios de inclusão adotados são os seguintes: ter diagnóstico de diabetes mellitus tipo 2, tempo de diagnóstico clínico do diabetes mellitus tipo 2 inferior a 10 anos, Índice de Massa Corporal (IMC) entre 25 e 35 kg/m2, apresentar glicemia de jejum < 250mg/dL, apresentar pressão arterial (PA): sistólica 140 mmHg e diastólica 100 mmHg. Os critérios de exclusão adotados serão: presença de complicações agudas ou crônicas do diabetes mellitus que possam ser prejudicadas pelo programa de exercício físico. O Programa de Exercício Físico será realizado duas vezes por semana (terças e quintas-feiras) das 07h as 09h, com duração de 12 semanas, sempre com a supervisão da pesquisadora responsável e acadêmicos treinados do Curso de Licenciatura em Educação Física da Universidade Federal do Tocantins. As aulas serão realizadas na Academia do idoso de Tocantinópolis. A progressão dos treinamentos será realizada

de acordo com as diretrizes da American College Academy of Medicine.

Objetivo da Pesquisa:

Objetivo Primário:

Analisar o uso de diferentes formas de exercício físico no tratamento do diabetes mellitus de diabéticos tipo II.

Objetivo Secundário:

Analisar associação entre sexo, idade, tempo de diagnóstico de diabetes, aparecimento de complicações crônicas e controle lipídico e glicêmico por meio do exercício aeróbico e de força;

Avaliação dos Riscos e Benefícios:

Riscos:

A realização de exercícios físicos por pessoas pertencentes a grupos especiais (obesos, hipertensos e diabéticos) pode gerar riscos devido à sua condição de saúde, portanto para participar do Programa de Exercício Físico para Diabéticos será realizada a medida de glicemia capilar e pressão arterial antes, durante e após cada sessão do exercício, todos os dias. Para manter os participantes dentro de uma faixa considerada segura para realização dos exercícios físicos e evitar eventos cardiovasculares, não realizarão a sessão de exercícios os participantes que apresentarem glicemia capilar > 200mg/dcl e pressão arterial >140/10mmHg. Estes participantes farão uma sessão de relaxamento à parte, de forma a obterem benefícios a saúde por meio desse relaxamento e evitarem danos à saúde pela prática indevida de exercícios físicos.

Benefícios:

Como benefícios, os participantes do Programa de Exercício Físico para Diabéticos terão a oportunidade de complementar o tratamento do diabetes mellitus com a prática de exercício físico e melhorar a saúde cardiovascular, metabólica e psicológica, uma vez que o uso de medicamentos hipoglicemiantes não oferece os benefícios fisiológicos, psicológicos e sociais que a prática de exercícios físicos em grupo pode oferecer.

Comentários e Considerações sobre a Pesquisa:

A população deste estudo serão pessoas com diabetes tipo 2, de ambos os sexos, atendidos pelas Unidades Básicas de Saúde da Cidade de Tocantinópolis/Tocantins/Brasil.Durante as visitas às UBS, será solicitado uma sala à parte, previamente limpa e arejada, para realização da coleta de dados. O tamanho da amostra foi determinado utilizando o programa estatístico GPower®. Foram considerados os seguintes parâmetros para cálculo da amostra com 4 grupos: tamanho de efeito

de 40%, poder da amostra em 95%, probabilidade do erro tipo 1 em 0,05 e probabilidade do erro tipo 2 em 20%. Para todos os cálculos foi considerado um intervalo de confiança de 95%. Com base nesses parâmetros, o tamanho mínimo da amostra foi determinado em 60, número que foi acrescido em 40% para compensar possíveis perdas e recusas, resultando em uma amostra de 84 participantes.Para recrutar a amostra, os pesquisadores visitarão a UBS nos dias de atendimento às pessoas com diabetes e convidarão pessoalmente esses indivíduos a participar do estudo. Os indivíduos que aceitarem participar do Programa de Exercício Físico para Diabéticos farão parte da amostra do estudo e, independente do grupo de exercício físico em que serão alocados, todos preencherão um Protocolo de Inscrição contendo informações sociodemográficas e informações sobre qualidade do sono, percepção de saúde, qualidade de vida relacionada à saúde, informações sobre o diabetes e prática anterior de exercícios físicos e de atividade física antes do início do Programa de Exercício Físico para Diabéticos (anexo 2). Para evitar dificuldades na leitura e compreensão do questionário, o mesmo será aplicado no formato de entrevista pelos pesquisadores previamente treinados e com procedimentos padronizados para usar linguagem acessível para os participantes. A entrevista será realizada numa sala à parte, previamente solicitada à direção da UBS. Todos os questionários foram unidos em um único protocolo para padronizar a entrevista (Anexo 2). Os participantes que aceitarem participar do estudo serão distribuídos randomicamente em diferentes grupos de exercício físico, que serão:

• Grupo de Exercício Aeróbio
• Grupo de Treinamento de Força
• Grupo Combinado (treinamento de força + aeróbio)
• Grupo Controle

A distribuição de participantes por grupo será às cegas, de forma que não será possível determinar a proporção de participantes por idade ou sexo em cada grupo. De acordo com o grupo para o qual foi designado, o participante seguirá um protocolo de treinamento do Programa de Exercícios Físicos para diabéticos. Para o treinamento do Grupo aeróbio, o exercício físico será prescrito a partir do teste de esforço ergométrico. Para o Grupo de Treinamento de Força, o exercício físico será prescrito conforme o teste de 1RM ou a falha concêntrica momentânea (de acordo com as condições de saúde do participante) seguindo uma série de exercícios de força. O teste de esforço será realizado para determinar a capacidade funcional basal. A intensidade do exercício físico no início do treinamento aeróbio, treinamento resistido, treinamento aeróbio + treinamento resistido, será de 40% da freqüência cardíaca de reserva (FCR) de acordo com o teste de esforço progredindo

para 80% da FCR. Os participantes do grupo controle receberão palestras e orientações para a prática de exercício físico ou sessões de exercícios de relaxamento sem progressão da intensidade, a fim de que se beneficiem também com este projeto.

O Programa será realizado duas vezes por semana (terças e quintas-feiras) das 07h as 09h, e terá a duração de 12 semanas, sempre com a supervisão da pesquisadora responsável e acadêmicos treinados do Curso de Licenciatura em Educação Física da Universidade Federal do Tocantins (UFT). Durante o preenchimento do protocolo de inscrição o participante será questionado sobre a prática de exercício físico regular anterior à participação no Programa de Exercício Físico para Diabéticos (Anexo 2, Item V). O nível de atividade física poderá ser avaliado usando a versão longa do IPAQ (International Physical Activity Questionnaire - IPAQ) (Anexo 2, Item VII), questionário já testado e validado no Brasil17. O IPAQ contém 5 questões divididas em 5 domínios de atividade física, que são: domínio 1 – atividade física no trabalho, domínio 2 – atividade física como meio de transporte, domínio 3 – atividade física em casa ou apartamento: trabalho, tarefas domésticas e cuidar da família, domínio 4 – atividades físicas de recreação, esporte, exercício e de lazer e domínio 5 – tempo gasto sentado.

Considerações sobre os Termos de apresentação obrigatória:

- Cronograma: Documento entregue, e apresenta-se adequado.
- Orçamento: Documento entregue e apresenta-se adequado.
- Folha de Rosto: documento entregue e devidamente preenchido.
- Informações Básicas: documento entregue apresenta-se adequado.
- TCLE: Foi apresentado estando adequado para a execução da pesquisa. Apresenta-se claro quanto ao objetivo, metodologia, benefícios da pesquisa, condição de sigilo, contatos dos pesquisadores, participação voluntária e possibilidade do indivíduo se retirar a qualquer momento da pesquisa.
- Autorização de execução da Pesquisa: Documento entregue e apresenta-se adequado.
- Declaração de fase inicial: Documento entregue e apresenta-se adequado.
- Declaração do orientador: Documento entregue e apresenta-se adequado.
- Instrumento:O instrumento está adequado.
- Carta de apresentação ao CEP: Documento entregue adequado

Conclusões ou Pendências e Lista de Inadequações:

Aprovado

Considerações Finais a critério do CEP:

Este parecer foi elaborado baseado nos documentos abaixo relacionados:

Tipo Documento	Arquivo	Postagem	Autor	Situação
Informações Básicas do Projeto	PB_INFORMAÇÕES_BÁSICAS_DO_PROJETO_698672.pdf	13/11/2016 10:57:44		Aceito
Projeto Detalhado / Brochura Investigador	PROJETO_diabetes_cep_6.doc	13/11/2016 10:56:44	Joana Marcela Sales de Lucena	Aceito
Cronograma	cronograma_6.pdf	13/11/2016 10:56:15	Joana Marcela Sales de Lucena	Aceito
Outros	instrumentodecoletadedados.pdf	21/10/2016 12:49:43	Joana Marcela Sales de Lucena	Aceito
TCLE / Termos de Assentimento / Justificativa de Ausência	TCLE.pdf	23/08/2016 14:06:10	Joana Marcela Sales de Lucena	Aceito
Outros	decla_fase_inic.pdf	15/06/2016 15:02:31	Joana Marcela Sales de Lucena	Aceito
Outros	carta_apresen_cep.pdf	15/06/2016 15:02:08	Joana Marcela Sales de Lucena	Aceito
Orçamento	orcamento.pdf	15/06/2016 15:00:12	Joana Marcela Sales de Lucena	Aceito
Outros	decla_orient.pdf	31/05/2016 19:33:28	Joana Marcela Sales de Lucena	Aceito
Declaração de Instituição e Infraestrutura	carta_anuencia.pdf	31/05/2016 19:26:34	Joana Marcela Sales de Lucena	Aceito
Folha de Rosto	folha_de_rosto.pdf	31/05/2016 19:17:46	Joana Marcela Sales de Lucena	Aceito

Situação do Parecer:
Aprovado

Necessita Apreciação da CONEP:
Não

PALMAS, 08 de Dezembro de 2016

Assinado por:
Patrick Letouze Moreira
(Coordenador)

Endereço: Avenida NS 15, 109 Norte Prédio do Almoxarifado
Bairro: Plano Diretor Norte **CEP:** 77.001-090
UF: TO **Município:** PALMAS
Telefone: (63)3232-8023 **E-mail:** cep_uft@uft.edu.br

Endereço: Avenida NS 15, 109 Norte Prédio do Almoxarifado
Bairro: Plano Diretor Norte **CEP:** 77.001-090
UF: TO **Município:** PALMAS
Telefone: (63)3232-8023 **E-mail:** cep_uft@uft.edu.br
